# An easy, rapid, and sensitive method for detection of drug-resistant influenza virus by using a sialidase fluorescent imaging probe, BTP3-Neu5Ac

**DOI:** 10.1371/journal.pone.0200761

**Published:** 2018-07-12

**Authors:** Daisuke Kato, Yuuki Kurebayashi, Tadanobu Takahashi, Tadamune Otsubo, Hitomi Otake, Mika Yamazaki, Chihiro Tamoto, Akira Minami, Kiyoshi Ikeda, Takashi Suzuki

**Affiliations:** 1 Department of Biochemistry, School of Pharmaceutical Sciences, University of Shizuoka, Shizuoka-shi, Shizuoka, Japan; 2 Department of Organic Chemistry, School of Pharmaceutical Sciences, Hiroshima International University, Kure-shi, Hiroshima, Japan; University of Rochester Medical Center, UNITED STATES

## Abstract

Immunochromatographic kits and RT-PCR are widely used as diagnostic tools for influenza detection in clinical and hygiene fields. Immunochromatographic kits are useful for differential typing of influenza A and influenza B but cannot show if the detected virus strains have acquired drug resistance against neuraminidase inhibitors that target sialidase activity of viral neuraminidase. Although RT-PCR enables determination of drug-resistant mutants, its efficacy is limited to viruses carrying a known substitution in their neuraminidase genome sequence. In the present study, an easy, rapid and sensitive method for detection of drug-resistant influenza viruses regardless of major antigenic changes or genomic mutations was developed. By using the method in combination with virus-concentrated membranes in centrifugal filter units and a sialidase imaging probe, 2-(benzothiazol-2-yl)-4-bromophenyl-*N*-acetylneuraminic acid (BTP3-Neu5Ac), sialidase activity of influenza neuraminidase was visualized on membranes by the green fluorescence of produced hydrophobic BTP3 under UV irradiation with a handheld UV flashlight. Fluorescence images in the presence or absence of neuraminidase inhibitors clearly discriminated drug-resistant influenza viruses from drug-sensitive ones. The assay can be done within 15 min. The detection sensitivity was shown to be equal to or higher than the sensitivities of commercial immunochromatographic kits. The assay will be a powerful tool for screening and monitoring of emerging drug-resistant influenza viruses and would help clinicians decide effective antiviral treatment strategies when such mutants have become prevalent.

## Introduction

Influenza virus is a major cause of severe and acute respiratory tract infections. Complications of influenza virus infection can result in viral pneumonia, secondary bacterial pneumonia, encephalopathy in infants, or sinusitis, which may lead to increased mortality, especially among people in high-risk groups [1–4]. Global spread of influenza virus affects up to 10% of the world’s population annually and accounts for more than 15 million cases of infection. There are about 15,000 hospitalizations in one influenza season in Japan alone [5,6]. Seasonal influenza epidemics that are attributed to influenza A virus (IAV) subtypes of H1N1pdm (new H1N1 that emerged during the influenza pandemic of 2009) and H3N2 in addition to influenza B virus (IBV) occur every year despite the global availability of influenza vaccines. There is a possibility that a new subtype of influenza virus will infect humans and trigger a pandemic that may cause serious health problems with social and economic impacts. Since the first reported case of human infection with avian IAV (H7N9) in 2013, the number of patients with H7N9 infection has been increasing, especially in China [7]. This suggests the pandemic potential of new subtypes of human IAV that originated in avian IAV. Furthermore, if a highly pathogenic avian H5N1 influenza virus spreads throughout the world, it could cause catastrophic pandemics due to its high mortality rate of approximately 60% [8]. Therefore, disease control with anti-influenza agents is of great importance for the prevention of potential pandemics. On the other hand, epidemic influenza infection, which is highly contagious, show rapid progression of symptoms, and has the potential to induce encephalopathy, especially in infants, should also be diagnosed at an early stage of disease onset and then appropriate medication with anti-influenza agents should be initiated as soon as possible [4,9].

Three classes of antiviral drugs have been shown to be effective against influenza virus infection: M2 ion channel blocker, neuraminidase inhibitor (NAI), and viral RNA polymerase inhibitor [10–12]. M2 ion channel blockers (amantadine and rimantadine) exhibit antiviral activity against IAV alone by inhibiting proton channel activity of viral M2 protein that is essential for the uncoating process of viral internal proteins during viral cell entry [13]. Since the majority of seasonal influenza viruses have already acquired resistance against M2 ion channel blockers, the use of these drugs for treatment of current IAV is no longer recommended [14]. NAIs target sialidase activity of viral neuraminidase, which plays an essential role at the release stage of progeny virus from the cellular surface. NAIs suppress virus replication by inhibiting sialidase activity of neuraminidase proteins of both IAV and IBV [15]. Four NAIs, oseltamivir (OV), peramivir (PV), zanamivir (ZV), and laninamivir (LV), have been developed and their efficacy against influenza infection has been evaluated [11,16,17]. The first influenza viral RNA polymerase inhibitor, favipiravir, has been found to have potent and selective inhibitory activity against influenza virus through direct interference in replication of the viral RNA genome [12]. In Japan, favipiravir has already been approved, but its use is permitted only when pandemic influenza viruses that are resistant to all of the conventional antiviral drugs have become prevalent. In the USA and Europe, favipiravir has been undergoing phase 3 clinical trials [18]. Therefore, NAIs are *de facto* standard anti-influenza drugs for treatment of influenza patients.

Over the past fifteen years, multiple cases of mutant IAV and IBV that have acquired resistance against NAIs have been reported worldwide [19–28]. Sustained monitoring of drug-resistant mutants and providing updated surveillance information on drug resistance to medical professionals are crucial measures for appropriate use of NAIs. Assay methods capable of detecting drug-resistant mutants will be beneficial for determination of effective antiviral drugs in clinical settings, especially in countries where the emergence of mutants is a concern due to the widespread use of NAIs for treatment or prophylaxis of influenza infection. At present, although commercial immunochromatographic kits are used as a first-choice diagnostic for influenza infection, these kits cannot show if the detected virus strains have acquired drug resistance against NAIs.

RT-PCR is generally the most sensitive and specific method for detection of drug-resistant and drug-sensitive influenza viruses. Although RT-PCR offers valuable insights into the known genome sequence of drug resistance, this approach is not suitable for detection of drug-resistant viruses with unknown mutations and is limited to a genome sequence of interest targeted by specific primers. Moreover, differences in the degree of NAI susceptibility cannot be identified by RT-PCR if multiple mutations have accumulated within one influenza virus strain. As an alternative approach to assess influenza virus sensitivity to NAIs, the synthetic sialidase substrates 2'-(4-methylumbelliferyl)-α-d-*N*-acetylneuraminic acid (4MU-Neu5Ac) and 1,2-dioxetane derivatives (NA-Star® and NA-XTD^TM^) have been widely used in laboratory experiments [29,30]. For analysis of sialidase localization mainly in mammalian cells, the synthetic sialidase substrate 5-bromo-4-chloro-3-indolyl-α-d-*N*-acetylneuraminic acid (X-Neu5Ac) and its fluorescing agent FastRed Violet LB (a fluorescing agent of X-Neu5Ac) have been used. Sialidase hydrolyzes *N*-acetylneuraminic acid (Neu5Ac)-linked chemical binding in the substrate structure to release fluorescent, chemiluminescent, and chromogenic products from 4MU-Neu5Ac, NA-Star®, and X-Neu5Ac, respectively. Since sialidase activity of drug-resistant viruses hydrolyzes the substrates even in the presence of NAIs, the use of such synthetic substrates enables assessment of drug resistance and susceptibility. The use of sialidase substrates is more advantageous than RT-PCR because a sialidase activity-based reaction can be applied to almost all strains of influenza viruses and known/unknown drug-resistant mutations regardless of major antigenic changes or genomic mutations. However, enzymatic reaction processes require a long incubation period before the test results are obtained for all of the three substrates. The sensitivity of X-Neu5Ac is much lower than that of 4MU-Neu5Ac, NA-Star® and NA-XTD^TM^. Sialidase measurement with these substrates requires a dedicated apparatus for detecting fluorescence and chemiluminescence. In addition, fluorescence reaction of X-Neu5Ac with FastRed Violet LB is less efficient at pH of more than 5, which is the commonly used pH range for measurement of influenza sialidase activity [31–34]. Because of such characteristics, assays based on the synthetic substrates have not been used in point-of-care testing (POCT) kits.

We have developed a novel sialidase substrate, BTP3-Neu5Ac, that enables an easy and rapid assay for sialidase detection [35]. By sialidase reaction, water-soluble BTP3-Neu5Ac produces BTP3, which is a crystalline, water-insoluble, acid-resistant, and fluorescently stable compound (Ex/Em = 372 nm/526 nm). Although BTP3-Neu5Ac itself is not fluorescent, BTP3 is readily deposited in a location of sialidase activity, enabling fluorescent imaging of dot-blotted virus and virus-infected cells. BTP3-Neu5Ac can visualize sialidase activity blotted on filter papers or polyvinylidene difluoride (PVDF) membranes since water-insoluble BTP3 is easily concentrated on the membranes after sialidase reaction [35,36]. BTP3-Neu5Ac is applicable to sialidase-carrying viruses such as IAV, IBV, Newcastle disease virus, Sendai virus, human parainfluenza virus, and mumps virus [36–40].

In a previous report, we described a new assay method for selective detection and isolation of an NAI-resistant IAV from live infected cells by reaction with BTP3-Neu5Ac in the presence of NAIs [41]. The time, work, and dedicated apparatus involved in this cell culture-based assay make it difficult for application of BTP3-Neu5Ac to a POCT kit for detecting NAI-resistant viruses in clinical practice and in hygiene surveys. However, it is expected that visualization of sialidase activity blotted on filter membranes with BTP3-Neu5Ac can be utilized as such a POCT kit. For the purpose of developing a BTP3-based POCT procedure to detect NAI-resistant viruses, we aimed to improve the sensitivity for detection of IAV and IBV by concentration of the virus by ultrafiltration with filter membranes and optimization of sialidase reaction conditions and to simplify the detection procedure, which would also shorten the measurement time. In the present study, we established an easy, rapid, and sensitive assay method that is suitable for a POCT kit for detection of NAI-resistant viruses. For two subtypes of IAV (H1N1pdm and H3N2) and two lineages of IBV (Yamagata and Victoria lineages) that have caused recent seasonal influenza epidemics, in addition to NAI-resistant IAV, detection sensitivity of the assay method was compared with that of commercial influenza immunochromatographic kits.

## Materials and methods

### Chemicals

BTP3-Neu5Ac was purchased from Wako Pure Chemical Industries, Ltd., Osaka, Japan. Four anti-influenza NAIs, OV (carboxylate), PV, ZV and LV, were purchased from Chemscene LLC, AdooQ BioScience, LKT Laboratories Inc., and Toronto Research Chemicals Inc., respectively. OV (carboxylate) was used in all experiments as an active form showing sialidase inhibitory activity.

### Commercial influenza immunochromatographic kits

Prorast® Flu One, QuickNavi^TM^-Flu, ESPLINE® Influenza A&B-N, Primecheck® Flu, Quick Chaser® Flu A, B, BD Flu Examen^TM^, QuickVueRapid SP influ, Fine Vision Influenza, ImmunoAce® Flu, GOLD SIGN FLU, Clearview Exact Influenza A&B, Nanotrap® Flu A•B, Immunotrap Influenza A•B, Alsonic® Flu, Immunofine^TM^ FLU, and Rapid Testa® color FLU stick were purchased from LSI Medience Co., Denka Seiken Co., Ltd., Fujirebio Inc., Alfresa Pharma Co., Mizuho Medy Co., Ltd., Nippon Becton Dickinson Company, Ltd., DS Pharma Biomedical Co., Ltd., Alere Medical Co., Ltd., Tauns Laboratories Inc., Morinaga Milk Industry Co., Ltd., Alere Medical Co., Ltd., ROHTO Pharmaceutical Co., Ltd., Wako Pure Chemical Industries, Ltd., Alfresa Pharma Co., Nichirei Biosciences Inc., and Sekisui Medical Co., Ltd., respectively.

### Cells, viruses, and bacteria

Cells were obtained from ATCC. Madin-Darby canine kidney (MDCK) cells (ATCC-CCL-34) were grown in minimum essential medium (MEM) supplemented with 5% fetal bovine serum (FBS). African green monkey kidney Vero cells (ATCC-CCL-81) were maintained in MEM supplemented with 5% FBS. LLC-MK_2_ cells (ATCC-CCL-7) were maintained in MEM supplemented with 5% FBS. IAV strain A/Puerto Rico/8/1934 (H1N1) was propagated in the allantoic sacs of 10-day-old embryonated eggs and purified by sucrose density gradient centrifugation as described previously [42]. IAV strain A/Memphis/1/1971 (H3N2) and IBV strain B/Lee/1940 were grown in MDCK cells. Mumps virus (MuV) was propagated in Vero cells. Human parainfluenza virus type 1 (HPIV-1) was propagated in LLC-MK_2_ cells. Hemagglutination units (HAU) of the viruses were determined as described previously [43]. HAU were expressed as the highest dilution of the virus suspension giving complete agglutination of guinea pig erythrocytes. The IAV strains A/Shizuoka/17/2016 (H1N1pdm), A/Shizuoka/19/2016 (H1N1pdm), A/Shizuoka/30/2014 (H1N1pdm), and A/Shizuoka/1573/2009 (H1N1pdm) and the IBV strains B/Shizuoka/7/2014 (Victoria lineage) and B/Shizuoka/95/2013 (Yamagata lineage) were provided by Shizuoka Institute of Environment and Hygiene. Both A/Shizuoka/30/2014 (H1N1pdm) and A/Shizuoka/1573/2009 (H1N1pdm) strains have a hystidine-to-tyrosine substitution at position 275 (H275Y, based on amino acid numbering of human IAV N1 neuraminidase) in the neuraminidase. *Streptococcus pneumoniae* (ATCC-49619) was purchased from ATCC.

### Effect of calcium ion on viral sialidase activity and its enzymatic thermostability

A/Puerto Rico/8/1934 (H1N1), A/Memphis/1/1971 (H3N2), and B/Lee/1940 were suspended in 100 mM acetate buffer (pH 6.0) supplemented with 1.0 to 500 mM CaCl_2_ to adjust HAU of each virus to 2^4^. BTP3-Neu5Ac was diluted in 100 mM acetate buffer (pH 6.0) supplemented with 1.0 to 500 mM CaCl_2_ to give a concentration of 200 μM. An equal volume of virus suspension and 200 μM BTP3-Neu5Ac were mixed in a microtube (0.2 ml PCR Tube with Flat Cap, Neptune Scientific, CA, USA) followed by incubation at 37, 50, or 60°C for 10 min with a dry heat block (MyMiniBLOCK, ATTO, Tokyo, Japan). One hundred microliters of the reaction mixture was transferred to a 96-well microtitre plate (Nunclon Delta Surface, Thermo Scientific, MA, USA), and fluorescence from sialidase reaction was quantified by a microplate reader (Infinite 200 multi-mode reader, Tecan, Männedorf, Switzerland) with wavelengths of excitation and emission at 372 nm/526 nm for BTP3.

### Comparison of temperature dependency of sialidase activity from three influenza virus strains

A/Puerto Rico/8/1934 (H1N1), A/Memphis/1/1971 (H3N2), and B/Lee/1940 were suspended in 100 mM acetate buffer (pH 6.0) supplemented with 50 mM CaCl_2_ to adjust HAU of each virus to 2^4^. BTP3-Neu5Ac was diluted in 100 mM acetate buffer (pH 6.0) supplemented with 50 mM CaCl_2_ to give a concentration of 200 μM. An equal volume of virus suspension and 200 μM BTP3-Neu5Ac were mixed in the microtube followed by incubation at 37, 50, 52, 54, 56, 58, 60, and 70°C for 10 min with the dry heat block. One hundred microliters of the reaction mixture was transferred to a 96-well microtitre plate, and fluorescence from sialidase reaction was quantified by the microplate reader.

### Effect of pH on viral and bacterial sialidase activities

Viruses and *S*. *pneumoniae* were each suspended in 100 mM acetate buffer (pH 4.0, 5.0, 5.5, 6.0, and 6.5) supplemented with 50 mM CaCl_2_. To give an equivalent sialidase activity between influenza viruses at pH 5.0, HAU of A/Puerto Rico/8/1934 (H1N1), A/Memphis/1/1971 (H3N2), and B/Lee/1940 were adjusted to 2^4^. Under a pH 4.0 condition, the amounts of MuV, HPIV-1, and *S*. *pneumoniae* were standardized to make sialidase activity equal to that of 2^4^ HAU influenza viruses which was determined at pH 5.0. Then HAU of MuV and HPIV-1 were set at 2^0^ and 2^2^, respectively. *S*. *pneumoniae* was diluted to give 1.38 x 10^7^ colony-forming units (cfu)/mL. BTP3-Neu5Ac was diluted in 100 mM acetate buffer (pH 4.0, 5.0, 5.5, 6.0, and 6.5) supplemented with 50 mM CaCl_2_ to give a concentration of 200 μM. Equal volumes of samples and 200 μM BTP3-Neu5Ac were mixed in the microtube followed by incubation at 56°C for 15 min with the dry heat block. One hundred microliters of the reaction mixture was transferred to a 96-well microtitre plate, and fluorescence from sialidase reaction was quantified by the microplate reader. For MuV, HPIV-1, and *S*. *pneumoniae*, sialidase activity (%) was indicated as a percentage of the enzymatic activity at pH 4.0. For IAV and IBV, sialidase activity at pH 5.0 was defined as 100%.

### Time course study and comparison of detection sensitivities in two experimental conditions

A/Puerto Rico/8/1934 (H1N1) was suspended in 100 mM acetate buffer (pH 6.0 or 6.5) supplemented with 1.0 or 50 mM CaCl_2_ to adjust HAU of each virus to 2^4^. BTP3-Neu5Ac was diluted in 100 mM acetate buffer (pH 6.0 or 6.5) supplemented with 1.0 or 50 mM CaCl_2_ to give a concentration of 200 μM. An equal volume of virus suspension and 200 μM BTP3-Neu5Ac were mixed in the microtube followed by incubation at 37°C for 10 min (pH 6.0) or 56°C for 15 min (pH 6.5) with the dry heat block. Fluorescent images of BTP3 were observed using a UV Transilluminator (UV BOX-W, NATURAL IMMUNITY, Tokyo, Japan) at 365 nm. One hundred microliters of the reaction mixture was transferred to a 96-well microtitre plate, and fluorescence from sialidase reaction was quantified by the microplate reader. HAU of the maximum dilution at which the sample showed RFU (relative fluorescence units) of >200 after blank subtraction were defined as the lower limit of detection.

### Assay principle and detection sensitivity of the BTP3-based filter method

A/Puerto Rico/8/1934 (H1N1) was serially diluted with 100 mM acetate buffer (pH 6.5) supplemented with 50 mM CaCl_2_, and 100 μL of the virus suspension was loaded onto centrifugal filter units (Nanosep® 300K Centrifugal Filter Device, PALL Co., NY, USA) followed by centrifugation at 2,000 *g* for 1 min with a benchtop centrifuge (Micro Six, AS ONE, Osaka, Japan). After centrifugation, 50 μL of 100 μM BTP3-Neu5Ac in 100 mM acetate buffer (pH 6.5) supplemented with 50 mM CaCl_2_ was directly added onto the filter membrane and the filter unit was incubated at 56°C for 15 min with the dry heat block (BTP3-based filter method). After the reaction, BTP3 deposited on the membrane was observed by the eye under UV irradiation with a handheld UV flashlight (PW-UV343H-03L, KONTEC Co., Ltd., Osaka, Japan) at 375 nm. Filter membranes were removed from the centrifugal units and inserted into a 96-well microtitre plate. Fluorescence intensity of BTP3 on of the membrane was quantified by a microplate reader (SpectraMax Gemini XS, Molecular Device, CA, USA) with wavelengths of excitation and emission at 372 nm/526 nm for BTP3. Upon reading, the multiple-point well scanning function (Pattern: Fill, Density: 3, Spacing: 1.1 mm, Total Points: 9) was activated and the average of RFU from nine different measurement points was calculated. HAU of the maximum dilution at which the sample showed RFU of >2,000 were defined as the lower limit of detection.

### Detection sensitivity of the BTP3-based filter method in comparison with that of ESPLINE® Influenza A&B-N

A/Puerto Rico/8/1934 (H1N1) was serially diluted with phosphate buffered saline (PBS) and detected with 16 commercial influenza immunochromatographic kits according to the instructions of the manufacturer in order to compare detection sensitivities among these kits. Since ESPLINE® Influenza A&B-N has been shown to be one of the most sensitive immunochromatographic kits for A/Puerto Rico/8/1934 (H1N1) detection, we selected ESPLINE® Influenza A&B-N as evaluation criteria of detection sensitivity. Nine virus strains, A/Puerto Rico/8/1934 (H1N1), A/Shizuoka/17/2016 (H1N1pdm), A/Shizuoka/19/2016 (H1N1pdm), A/Shizuoka/30/2014 (H1N1pdm), A/Shizuoka/1573/2009 (H1N1pdm), A/Memphis/1/1971 (H3N2), B/Lee/1940, B/Shizuoka/7/2014 (Victoria lineage), and B/Shizuoka/95/2013 (Yamagata lineage), were serially diluted with 100 mM acetate buffer (pH 6.5) supplemented with 50 mM CaCl_2_ and assayed by the BTP3-based filter method. Fluorescence intensity of BTP3 on the membrane was quantified by a microplate reader (SpectraMax Gemini XS), and the maximum dilution at which the sample showed RFU of >2,000 was defined as the lower limit of detection for the BTP3-based filter method. For ESPLINE® Influenza A&B-N, viruses were diluted with extraction buffer of the kit. Twenty microliters of the sample was spotted on the test device and left for 15 min at room temperature. Test results were evaluated by the naked eye according to the manufacturer’s instructions. The maximum dilution required for a positive reaction was determined as the lower limit of detection for ESPLINE® Influenza A&B-N. Detection sensitivity of each virus strain in the BTP3-based filter method was compared as relative ratio (fold change) of that of each virus with ESPLINE® Influenza A&B-N.

### Detection of drug-resistant viruses in the BTP3-based filter method

Viruses and *S*. *pneumoniae* were each suspended in 100 mM acetate buffer (pH 6.5) supplemented with 50 mM CaCl_2_. To give an equivalent sialidase activity, the amounts of viruses and *S*. *pneumoniae* were standardized as described in the ‘Effect of pH on viral and bacterial sialidase activities’ section. OV, PV, ZV, and LV were each diluted with 100 mM acetate buffer (pH 6.5) supplemented with 50 mM CaCl_2_ to give a concentration of 2.0 μM. NAI-supplemented BTP3-Neu5Ac was prepared by mixing 200 μM BTP3-Neu5Ac and 2.0 μM of each NAI at a ratio of 1:1. One hundred microliters of the virus suspension or *S*. *pneumoniae* suspension was loaded onto centrifugal filter units and centrifuged at 2,000 *g* for 1 min with the benchtop centrifuge. After centrifugation, 50 μL of NAI-supplemented BTP3-Neu5Ac or 100 μM BTP3-Neu5Ac was directly dropped onto the filter membranes. Then the filter units were incubated at 56°C for 15 min with the dry heat block. BTP3 deposited on the membrane was observed by the eye under UV irradiation with the handheld UV flashlight at 375 nm.

## Results

### Effect of calcium ion on viral sialidase activity and its enzymatic thermostability

**Fig 1:**
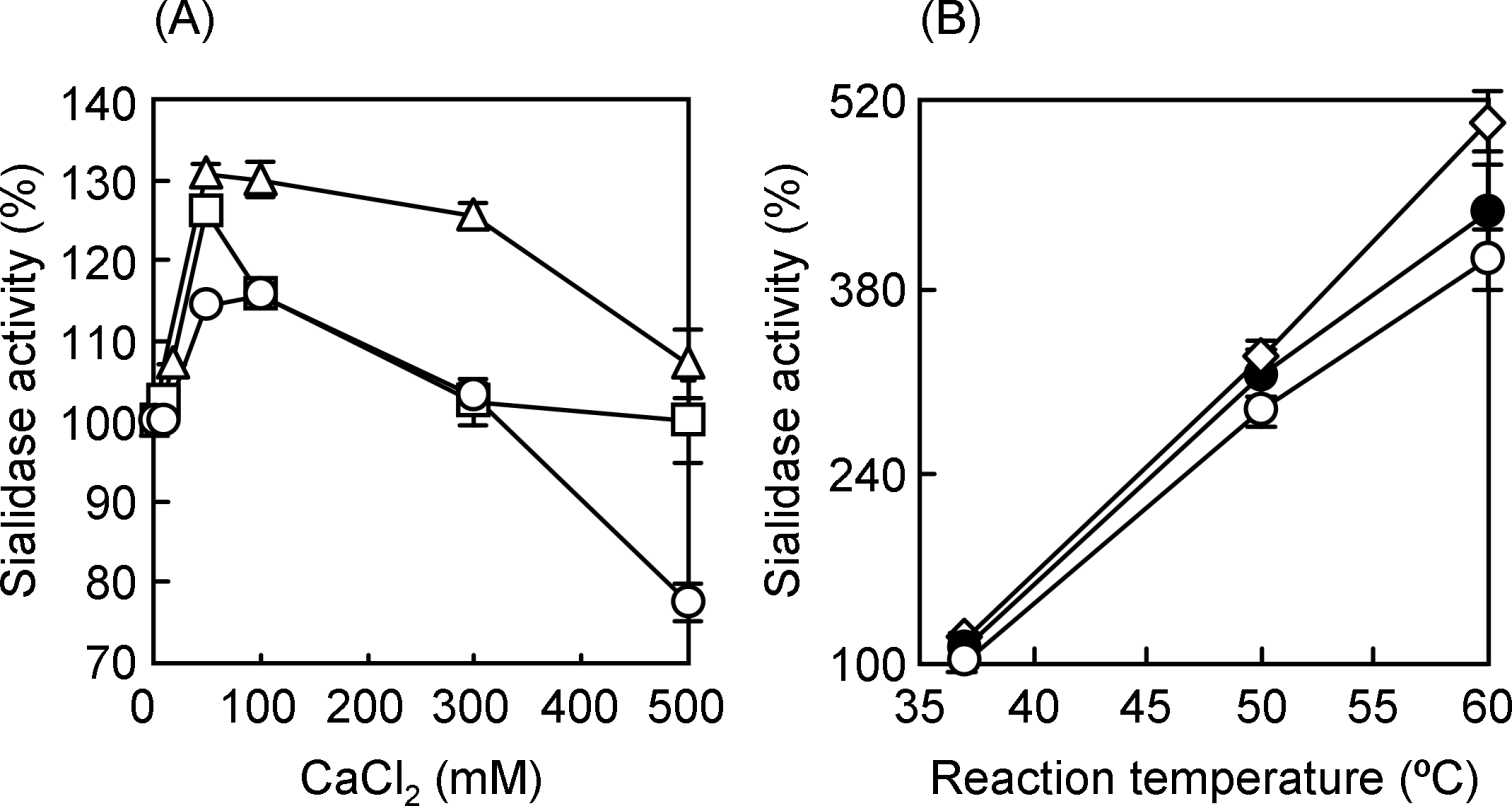
Effect of calcium ion on viral sialidase activity and its enzymatic thermostability. (A) A/Puerto Rico/8/1934 (open circles), A/Memphis/1/1971 (open triangles), B/Lee/1940 (open squares), and BTP3-Neu5Ac were diluted in 100 mM acetate buffer (pH 6.0) supplemented with 1.0 to 500 mM CaCl_2_. An equal volume of virus suspension and 200 μM BTP3-Neu5Ac were mixed in a microtube followed by incubation at 37°C for 10 min. The reaction mixture was transferred to a 96-well microtitre plate and fluorescence intensity was read with a microplate reader. Sialidase activity (%) was expressed as a percentage of the activity in the sample with 1.0 mM CaCl_2_. Each value is the mean and standard deviation from at least three independent measurements. (B) Thermostability of A/Puerto Rico/8/1934 sialidase was assessed in the presence of 1.0, 25, or 50 mM CaCl_2_. A virus suspension was reacted with BTP3-Neu5Ac as in (A) except for the setting of reaction temperature at 37, 50, or 60°C. Sialidase activity (%) is expressed as a percentage of the activity in the experiment conducted at 37°C. Each value is the mean and standard deviation from at least three independent measurements. Open circles, closed circles, and open rhombuses represent 1.0, 25, and 50 mM CaCl_2_, respectively.

We investigated the optimal reaction conditions for sialidase activity to improve detection sensitivity of the BTP3-Neu5Ac reaction. Among divalent cations, calcium ion is essential for influenza sialidase activity and is responsible for thermostability of the enzymatic activity [44,45]. Optimization of the concentration of calcium ion and incubation temperature should increase the detection sensitivity of sialidase activity with BTP3-Neu5Ac. In order to examine the optimal concentration of calcium ion and the optimal temperature for BTP3-Neu5Ac reaction, we incubated IAV or IBV with 100 μM BTP3-Neu5Ac under varying conditions of CaCl_2_ concentration and temperature. Sialidase activity was compared with that in the original experimental condition, 37°C incubation for 10 min in the presence of 1.0 mM CaCl_2_, as described in our previous reports [36,41]. Viral sialidase activity was increased up to 1.3 fold at 50 mM CaCl_2_ (Fig 1A). To evaluate the thermostabilizing effect of calcium ion, we performed BTP3-Neu5Ac reaction at 37, 50, or 60°C in the presence of 1.0, 25, or 50 mM CaCl_2_. Viral sialidase activity of A/Puerto Rico/8/1934 markedly increased even under the condition of 60°C in a dose-dependent manner of CaCl_2_ (Fig 1B). The results indicated that 50 mM CaCl_2_ was an optimal concentration for BTP3-Neu5Ac reaction and that a high incubation temperature was an important factor to achieve maximum enzymatic activity.

### Comparison of the temperature dependencies of sialidase activities of three influenza virus strains

**Fig 2:**
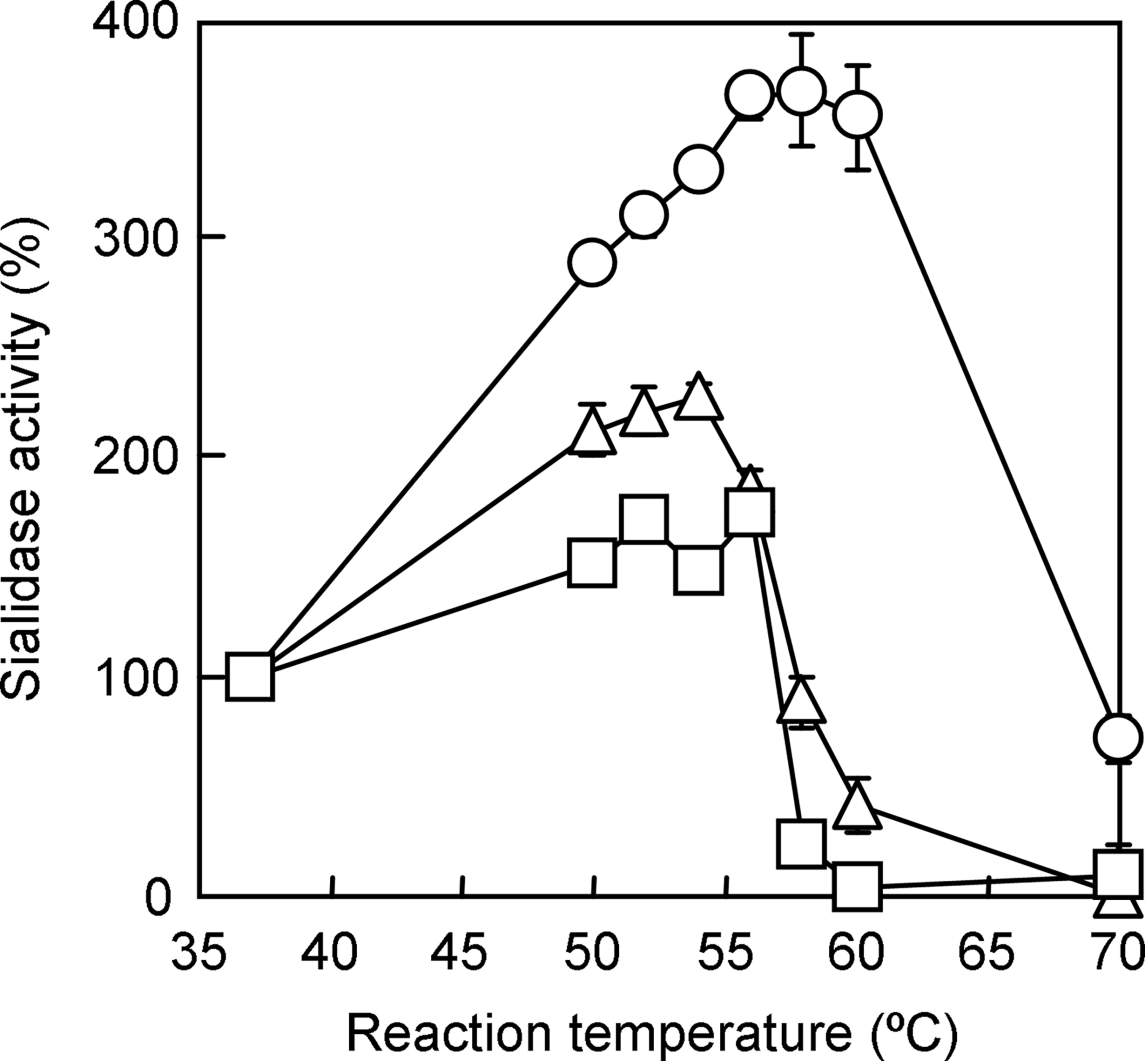
Comparison of the temperature dependencies of sialidase activities of three influenza virus strains. The temperature dependencies of sialidase activities of three influenza virus strains were compared. Viruses and BTP3-Neu5Ac were diluted in 100 mM acetate buffer (pH 6.0) supplemented with 50 mM CaCl_2_. An equal volume of virus suspension and 200 μM BTP3-Neu5Ac were mixed in a microtube followed by incubation at 37 to 70°C for 10 min. The reaction mixture was transferred to a 96-well microtitre plate and fluorescence intensity was read with a microplate reader. Sialidase activity (%) was expressed as a percentage of the activity in the experiment conducted at 37°C. Each value is the mean and standard deviation from at least three independent measurements. Open circles, open triangles, and open squares represent A/Puerto Rico/8/1934, A/Memphis/1/1971, and B/Lee/1940, respectively.

Although a high incubation temperature of 60°C contributed to a marked increase in sialidase activity and improved detection sensitivity (Fig 1B), it has been reported that there were differences in the temperature-activity profile and thermostability between sialidase activities of IAV and IBV [46]. Increased sialidase activity in a high temperature condition needs to be confirmed for IAV (H3N2) and IBV, as well as A/Puerto Rico/8/1934. In order to examine the optimal incubation temperature for BTP3-Neu5Ac reaction, we incubated IAV and IBV with 100 μM BTP3-Neu5Ac in the temperature range from 37 to 70°C in the presence of 50 mM CaCl_2_ for 10 min. As shown in Fig 2, within the range of 37 to 60°C, sialidase activity of A/Puerto Rico/8/1934 was temperature-dependently increased, being consistent with the data shown in Fig 1B. On the other hand, for A/Memphis/1/1971 and B/Lee/1940, the sialidase activities were greatly decreased at a temperature higher than 58°C. These results suggested that 56°C, but not 60°C, was the most appropriate temperature to enable sialidase detection of both IAVs (H1N1 and H3N2) and IBV.

### Discrimination of influenza sialidase activity from false-positive reaction caused by viral and bacterial sialidases

In addition to influenza virus, some paramyxoviruses and bacteria are known to possess sialidase activity. Among them, MuV, HPIV-1, and *S*. *pneumoniae*, all of which are human pathogens, express proteins with sialidase activity to facilitate budding of the infectious virions or infection to host cells [47,48]. MuV and HPIV-1 are known to coinfect with IAV, and *S*. *pneumoniae* is the most frequent coinfecting bacterium that causes morbidity and mortality in influenza patients [1,49,50]. MuV, HPIV-1, and *S*. *pneumoniae* can be causes of false-positive at diagnosis of influenza virus based on BTP3-Neu5Ac reaction. To discriminate influenza virus from possible false-positives in terms of reaction pH, we evaluated pH profiles of viral and bacterial sialidases (Table 1). For MuV and HPIV-1, the pH value at which sialidase activity was maximum was 4.0 and the sialidase activities decreased in a pH-dependent manner. Conversely, for *S*. *pneumoniae*, the sialidase activity exhibited 3-fold greater activation at pH 6.5 than that at pH 4.0. For IAV and IBV, the sialidase activities also increased in a pH-dependent manner.

**Table 1:** Effect of pH on viral and bacterial sialidase activities.

Sample	Reaction pH				
	4.0	5.0	5.5	6.0	6.5
MuV	100%	23%	40%	15%	7.6%
HPIV-1	100%	76%	36%	11%	-2.2%
*S*. *pneumoniae*	100%	54%	25%	313%	333%
A/Puerto Rico/8/1934 (H1N1)	-0.7%	100%	159%	182%	193%
A/Memphis/1/1971 (H3N2)	1.0%	100%	472%	650%	646%
B/Lee/1940	0.2%	100%	145%	168%	175%

Viruses or *S*. *pneumoniae* were incubated with 100 μM BTP3-Neu5Ac at 56°C in the presence of 50 mM CaCl_2_ for 10 min under different pH conditions. For MuV, HPIV-1, and *S*. *pneumoniae*, sialidase activity (%) was expressed as a percentage of the activity in the experiment conducted at pH 4.0. For IAV and IBV, sialidase activity under a pH 5.0 condition was defined as 100% since the enzymatic activity was extremely low at pH 4.0.

As the reaction pH was increased, sialidase activities of IAV, IBV, and *S*. *pneumoniae* were elevated, while the sialidase activity of HPIV-1 was completely lost. MuV also showed a pH-dependent decrease in its sialidase activity, although it was still active at pH 6.5. These results indicated that false-positive reactions that might be caused by HPIV-1 could be avoided by selecting reaction pH of 6.5 without interfering with influenza sialidase activity.

### Time course study and comparison of detection sensitivities in two experimental conditions

**Fig 3:**
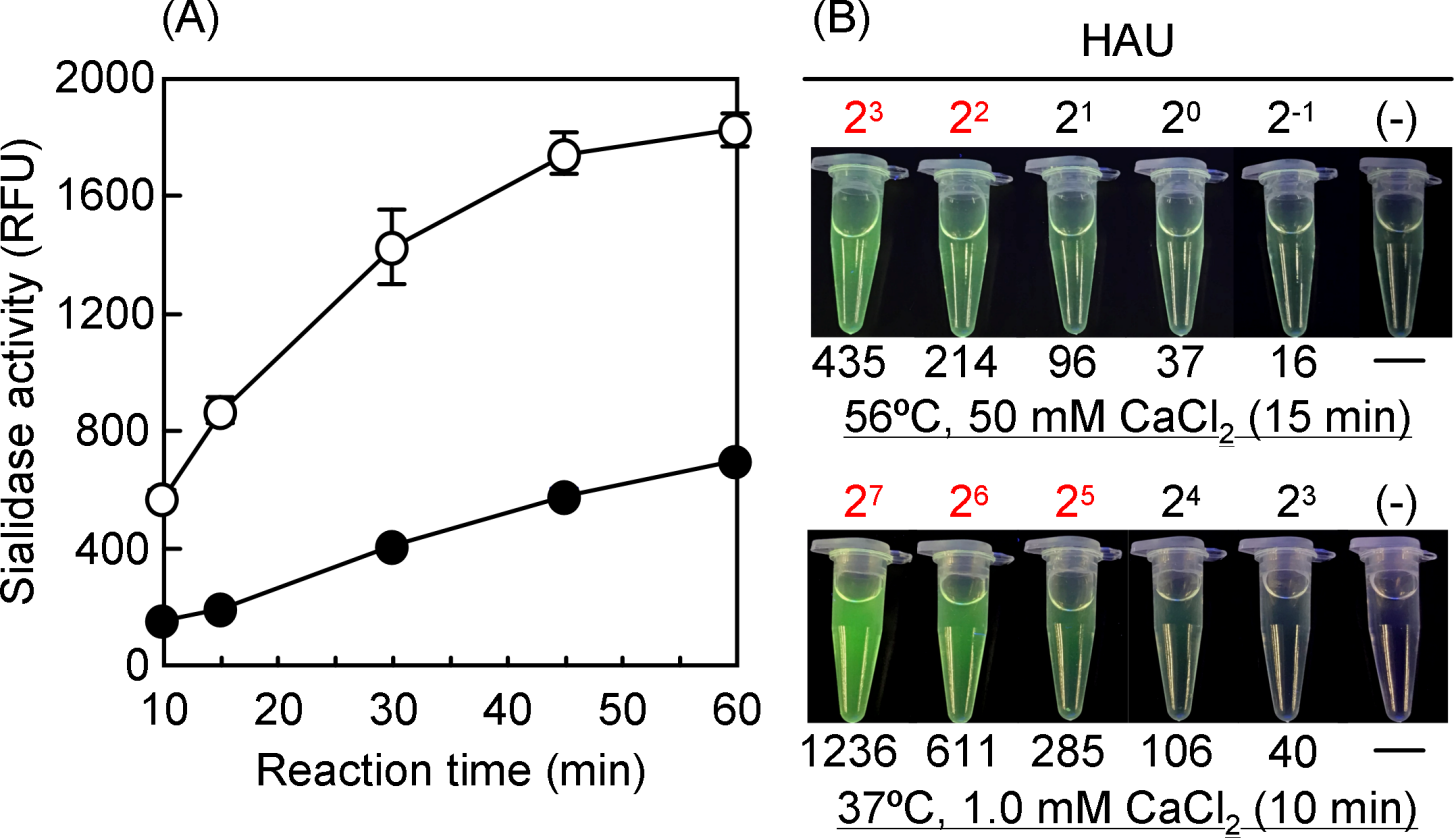
Time course study and comparison of detection sensitivities in two experimental conditions. (A) A/Puerto Rico/8/1934 was incubated with 100 μM BTP3-Neu5Ac under two different experimental conditions: one is the original experimental protocol based on our previous reports (37°C incubation in the presence of 1.0 mM CaCl_2_ at pH 6.0, closed circles) and the other is the optimized conditions (56°C incubation in the presence of 50 mM CaCl_2_ at pH 6.5, open circles). Fluorescence of the sialidase product, BTP3, was expressed as RFU and plotted against incubation time. Each value is the mean and standard deviation from at least three independent measurements. (B) A two-fold serial dilution of A/Puerto Rico/8/1934 was prepared and reacted with BTP3-Neu5Ac under the two different conditions described in (A) in microtubes. Fluorescent images of BTP3 were observed under UV irradiation at 365 nm. Samples were collected and transferred to a 96-well microtitre plate and fluorescence intensity was read with a microplate reader. RFU are indicated below the images of microtubes. Among the reaction mixtures, RFU of >200 after blank subtraction were considered to be positive results. HAU of the maximum dilution at which the sample was positive was defined as the lower limit of detection. Red letters indicate HAU of positive results. (-) indicates BTP3-Neu5Ac reaction in the absence of a virus and was used as a blank.

We performed a time course study to determine the appropriate incubation time for BTP3-Neu5Ac reaction (Fig 3A). Sialidase activity of A/Puerto Rico/8/1934 produced BTP3 from BTP3-Neu5Ac in a time-dependent manner until 60 min of the reaction end point to reach maximum fluorescent intensity of BTP3. Since long testing turnaround time is not preferred in rapid laboratory diagnostics treating numerous samples and since almost all commercial influenza immunochromatographic kits generally set the testing period within 15 min, we decided to choose 15 min for BTP3-Neu5Ac reaction to provide a rapid assay procedure that is comparable to that of the immunochromatographic kits. In the present optimized experimental protocol with 50 mM CaCl_2_ (pH 6.5) for 15 min at 56°C, the sialidase activity (871 RFU) was 5.7-times higher than that (153 RFU) in the previous experimental protocol with 1.0 mM CaCl_2_ (pH 6.0) for 10 min at 37°C. To visually assess detection sensitivity in the optimized conditions, two-fold serial dilutions of A/Puerto Rico/8/1934 were incubated with BTP3-Neu5Ac at 56°C for 15 min. Fluorescence images were observed under UV irradiation and fluorescence intensity of each reaction was quantified (Fig 3B). HAU of the maximum dilution at which the sample showed RFU of >200 after blank subtraction were defined as the lower limit of detection. The lower limits of detection in the present optimized protocol and the previous protocol were 2^2^ HAU and 2^5^ HAU, respectively. Detection sensitivity in the present optimized protocol became 8-fold higher than that in the previous protocol. However, we considered that detection sensitivity of 2^2^ HAU was not a sufficient level because some commercial influenza immunochromatographic kits were able to detect HAU of less than 2^−2^ for the same IAV (data not shown). Further improvement in detection sensitivity is required to achieve performance equivalent to that of commercial influenza immunochromatographic kits.

### Establishment of the BTP3-based filter method

To improve detection sensitivity of the BTP3-Neu5Ac reaction, we aimed to establish a BTP3-based filter method in which molecular cut-off ultrafiltration membranes are used for virus concentration and on-membrane virus is detected with BTP3-Neu5Ac reaction. To select an appropriate filter pore size that can efficiently trap influenza virus particles, a A/Puerto Rico/8/1934 suspension was centrifuged with 30 kDa, 100 kDa, and 300 kDa molecular weight cut-off filters, followed by measurement of viral sialidase activity in the flow-through (S1 Table). Sialidase activity was not recovered from the flow-through regardless of the filter pore sizes tested. The absence of enzymatic activity in the flow-through after ultrafiltration indicates that almost all of the influenza virus particles were trapped on the filter membranes. For the BTP3-based filter method, the largest pore size of 300 kDa was selected due to efficient filterability of virus samples.

**Fig 4:**
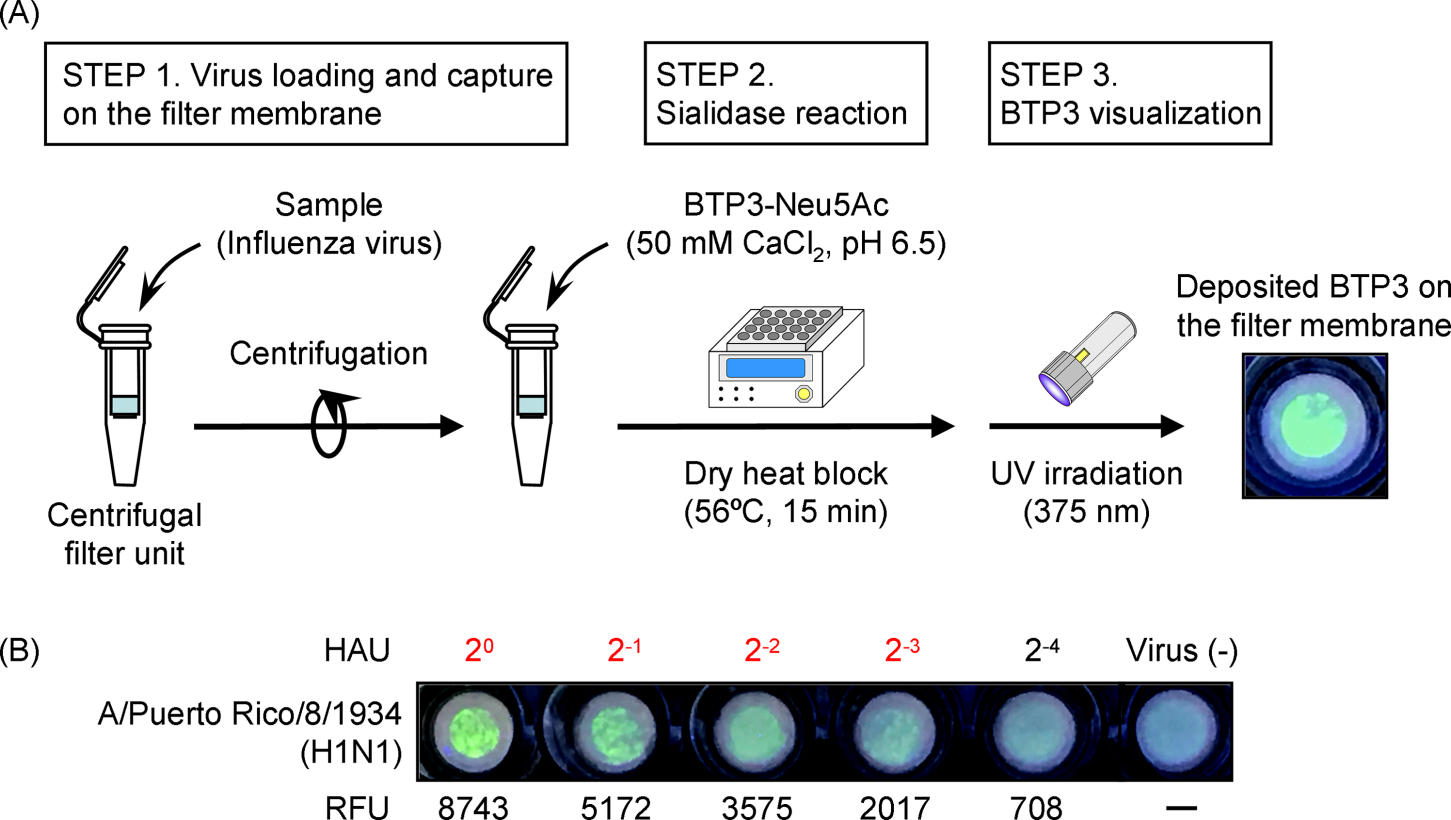
Assay principle and detection sensitivity of the BTP3-based filter method. (A) A virus solution was loaded onto filter units followed by centrifugation (STEP 1). Then BTP3-Neu5Ac was directly added onto the filter membrane and the filter units were incubated at 56°C for 15 min with a dry heat block (STEP 2). After the reaction, crystalline BTP3 released from BTP3-Neu5Ac by viral sialidase activity was deposited on the membrane. Fluorescent images of BTP3 were visualized under UV irradiation with a handheld UV flashlight (STEP 3). (B) Two-fold serial dilution of A/Puerto Rico/8/1934 was assayed by the BTP3 method and fluorescence intensity on the membrane was scanned with a microplate reader. RFU of >2,000 were considered to be positive results. HAU of the maximum dilution at which the sample was positive was defined as the lower limit of detection. Red letters indicate HAU of positive results. Virus (-) indicates BTP3-Neu5Ac reaction in the absence of a virus and was used as a blank.

A detailed outline of the BTP3-based filter method is as follows. A virus solution was concentrated by 1-min centrifugation using the centrifugal filter unit with a 300 kDa molecular cut-off membrane, and the virus particles were trapped on the surface of the membrane. BTP3-Neu5Ac in 100 mM acetate buffer supplemented with 50 mM CaCl_2_ (pH 6.5) was directly dropped onto the virus-trapped membrane followed by incubation at 56°C for 15 min with the dry heat block. Then water-insoluble BTP3 deposited on the membrane was visualized under UV irradiation with the handheld UV flashlight at 375 nm and fluorescent images were observed (Fig 4A). In order to confirm the detection sensitivity, two-fold serial dilutions of A/Puerto Rico/8/1934 were subjected to the BTP3-based filter method as outlined in Fig 4A. After the BTP3-Neu5Ac reaction, the filter membranes were removed from the centrifugal units and inserted into a 96-well microtitre plate. Fluorescence intensity of BTP3 on the membrane was quantified by the microplate reader. HAU of the maximum dilution at which the sample showed RFU of >2,000 were defined as the lower limit of detection. The BTP3-based filter method successfully detected a 2^−1^ HAU sample, which was hardly detectable in the experiment for which results are shown in Fig 3B. The minimum HAU detected in the BTP3-based filter method were proved to be 2^−3^. For A/Puerto Rico/8/1934, detection sensitivity in the BTP3-based filter method was 32-times higher than that in the present optimized protocol without centrifugal filter treatment (Fig 4B). Next, we evaluated the detection sensitivity of A/Puerto Rico/8/1934 by sixteen influenza immunochromatographic kits commercially available in Japan that do not need dedicated apparatuses. Among the kits tested, we singled out ESPLINE® Influenza A&B-N, which was shown to be one of the most sensitive kits for A/Puerto Rico/8/1934 detection (data not shown). We compared the lower limits of detection of ESPLINE® Influenza A&B-N and the BTP3-based filter method for IAV strains (H1N1, H3N2, and four H1N1pdm clinical isolates from the 2009–2016 influenza season) and IBV strains including Victoria and Yamagata lineages. Two-fold serial dilutions of the viruses were subjected to both ESPLINE® Influenza A&B-N and the BTP3-based filter method. Both methods took 15 min for incubation. The lowest detectable dilution rate of the viruses for each method was measured and expressed as fold change compared to ESPLINE® Influenza A&B-N (Table 2). The lowest detectable dilution rate was 0.5 to 8-fold higher in the BTP3-based filter method than in ESPLINE® Influenza A&B-N. Detection sensitivity in the BTP3-based filter method was equal to or higher than that in ESPLINE® Influenza A&B-N for all of the strains tested regardless of IAV, IBV, virus subtypes, and lineages.

**Table 2:** Detection sensitivity of the BTP3-based filter method in comparison with that of ESPLINE® Influenza A&B-N.

Influenza virus strain	The lowest detectable dilution rate of the viruses		Fold change compared to ESPLINE® Influenza A&B-N
	BTP3-based filter method	ESPLINE® Influenza A&B-N	
A/Puerto Rico/8/1934 (H1N1)	2^−14^	2^−12^	4.0
A/Shizuoka/17/2016 (H1N1pdm)	2^−15^	2^−15^	1.0
A/Shizuoka/19/2016 (H1N1pdm)	2^−14^	2^−14^	1.0
A/Shizuoka/30/2014 (H1N1pdm)	2^−18^	2^−16^	4.0
A/Shizuoka/1573/2009 (H1N1pdm)	2^−17^	2^−14^	8.0
A/Memphis/1/1971 (H3N2)	2^−19^	2^−17^	4.0
B/Lee/1940	2^−14^	2^−14^	1.0
B/Shizuoka/7/2014 (Victoria lineage)	2^−10^	2^−10^	1.0
B/Shizuoka/95/2013 (Yamagata lineage)	2^−10^	2^−11^	0.5

Two-fold serially diluted viruses were subjected to both ESPLINE® Influenza A&B-N and the BTP3-based filter method. The detectable virus dilution rate for each method was determined and expressed as a fold change compared to ESPLINE® Influenza A&B-N.

### Detection of drug-resistant viruses in the BTP3-based filter method

**Fig 5:**
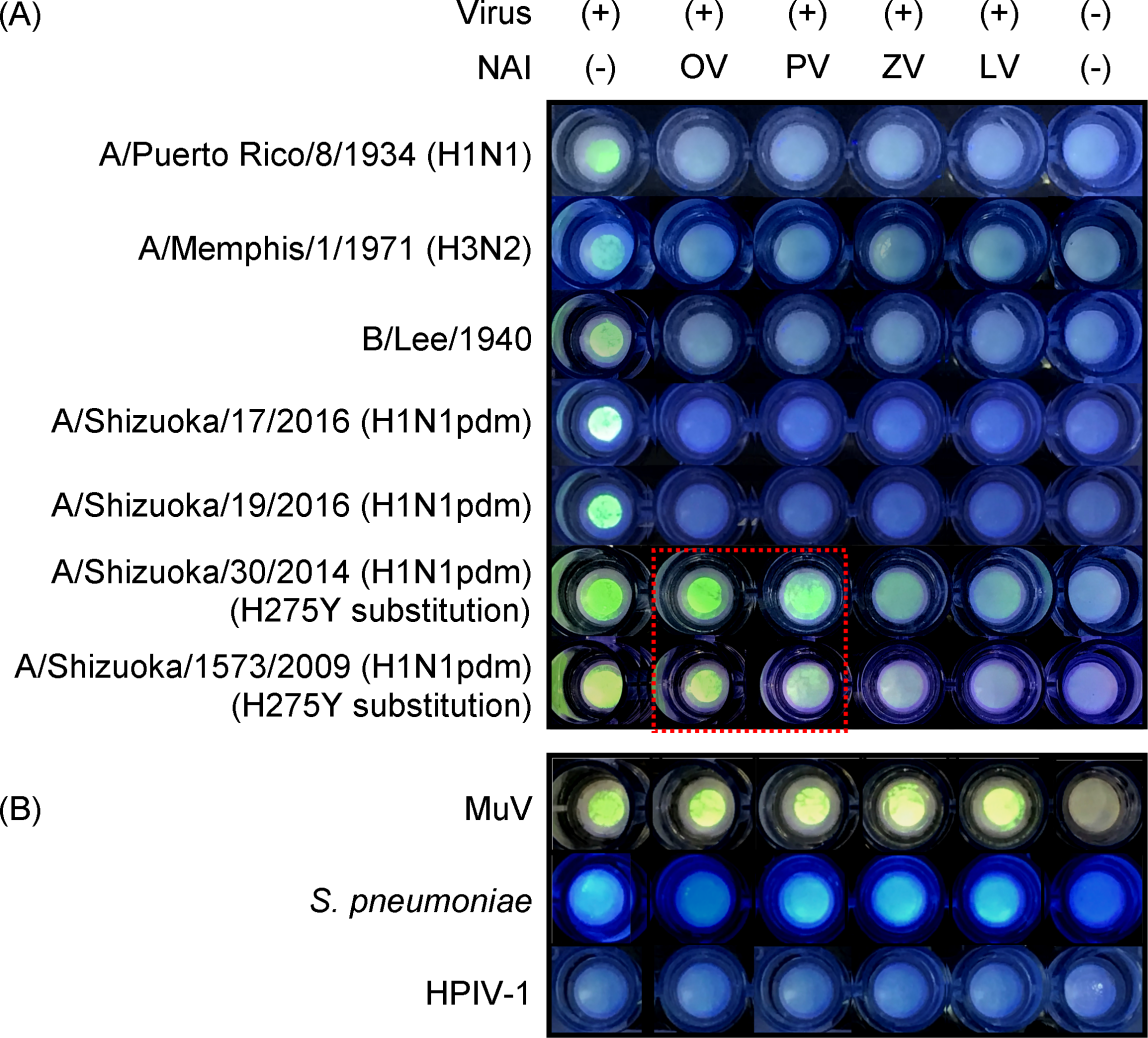
Detection of drug-resistant viruses in the BTP3-based filter method. (A) 100 μM BTP3-Neu5Ac supplemented with or without 10 μM NAIs was added directly onto the influenza virus-loaded filter membranes. After 15 min at 56°C, BTP3 was visualized with a handheld UV flashlight and NAI susceptibility was evaluated by comparing the fluorescent images for NAI (-) and NAI-supplemented samples. NAI susceptibility of seven influenza virus strains was assessed for each NAI. (B) Fluorescence patterns of agents that may cause false-positive results. MuV, *S*. *pneumoniae*, and HPIV-1 with known titres were loaded onto the filter membranes and BTP3-Neu5Ac reaction was carried out as described in (A). OV, PV, ZV, and LV represent oseltamivir, peramivir, zanamivir, and laninamivir, respectively.

Drug-resistant viruses can retain sialidase activity even with the amount of an NAI that inhibits sialidase activity of drug-sensitive viruses. We have reported selective detection and selective isolation of drug-resistant viruses at the levels of virus culture and infected cells by BTP3-Neu5Ac reaction in the presence of NAIs [41]. It is expected that the BTP3-based filter method will also be able to detect such resistant viruses. Six IAV strains (including two OV-resistant strains) and one IBV strain were tested in the BTP3-based filter method (Fig 5A). NAI concentration in BTP3-Neu5Ac solution was set at 1.0 μM because that concentration completely inhibited sialidase activity of drug-sensitive strains tested. In the presence of each NAI (OV, PV, ZV and LV), BTP3 fluorescence on the membrane was not observed without exception for A/Puerto Rico/8/1934, A/Memphis/1/1971, A/Shizuoka/17/2016, A/Shizuoka/19/2016, and B/Lee/1940. Conversely, A/Shizuoka/30/2014 and A/Shizuoka/1573/2009 with the most prevalent OV-resistant H275Y substitution (based on amino acid numbering of human IAV N1 neuraminidase protein) exhibited strong fluorescence even in the presence of OV and PV during BTP3-Neu5Ac reaction (dashed red line in Fig 5A). Indeed, the BTP3-based filter method was able to detect OV and PV resistances of OV-resistant IAV strains with H275Y substitution. These results suggest that in combination with NAIs, the BTP3-based filter method is able to clearly discriminate drug-resistant strains from drug-sensitive strains for respective NAIs.

As new NAI resistance of A/Shizuoka/30/2014 and A/Shizuoka/1573/2009, PV resistance was detected in the BTP3-based filter method. We tried to confirm NAI resistances of these strains by 4MU-Neu5Ac used in the conventional method for NAI susceptibility. For A(H1N1pdm) clinical isolates, A/Shizuoka/17/2016, A/Shizuoka/19/2016, A/Shizuoka/30/2014 and A/Shizuoka/1573/2009, the concentration of the four NAIs that reduced viral sialidase activity by 50% relative to a control mixture without NAIs was determined as 50% inhibitory concentration (IC_50_) (S2 Table). Two drug-sensitive strains, A/Shizuoka/17/2016 and A/Shizuoka/19/2016, showed low IC_50_ values of <1.0 nM for all of the NAIs. Two drug-resistant strains, A/Shizuoka/30/2014 and A/Shizuoka/1573/2009, showed greatly increased IC_50_ values for both OV and PV, which means 820 to 9730-fold reduced inhibition compared to the drug-sensitive strains.

It is expected that false-positives caused by HPIV-1 can be circumvented because of loss of its sialidase activity at pH 6.5. However, MuV and *S*. *pneumoniae* can be detected as false-positive because their sialidase activities can be detected under pH 6.5 (Table 1). To distinguish between positive and false-positive reactions by NAI inhibition patterns, we investigated NAI susceptibility of MuV and *S*. *pneumoniae* in the BTP3-based filter method (Fig 5B). MuV was not susceptible to the four NAIs and showed fluorescence in the presence of all of the NAIs. Unexpectedly, *S*. *pneumoniae* was susceptible only to OV and showed fluorescence in the presence of PV, ZV, and LV. As expected for HPIV-1, there was no fluorescence in the presence of any of the NAIs and BTP3-Neu5Ac.

### Evaluation of long storage stability of BTP3 fluorescence

To evaluate the storage stability of BTP3 fluorescence, we preserved a filter membrane after the BTP3-based filter method for 28 days in a light-shaded environment (S1 Fig). BTP3 fluorescence was stably maintained even after 28 days with no increase in background fluorescence.

## Discussion

In the present study, we established a BTP3-based filter method that enables detection of influenza viruses and their NAI resistances in an easy, rapid, and sensitive manner. Using centrifugal filter units, virus concentration and sialidase fluorescence imaging can be done on a single membrane with simple two-step processes. Water-insoluble fluorescent BTP3, which is a sialidase reaction product of BTP3-Neu5Ac, is readily deposited on the surface of the virus-loaded membrane. Without the need for any dedicated apparatuses, green fluorescent images of BTP3 were easily observed by the eye under UV irradiation with a handheld UV flashlight (Fig 4). The BTP3-based filter method enabled influenza virus detection with a sensitivity equivalent to or higher than the sensitivities of commercial influenza immunochromatographic kits (Table 2) and enabled simultaneous assessment of virus susceptibility to four NAIs.

The activity and thermostability of influenza virus sialidase is known to be dependent on the presence of calcium ions [44]. As described in previous reports [44,45], we confirmed a calcium ion concentration-dependent increase in sialidase activity of both IAV (H1N1, H3N2) and IBV. The temperature-activity profile of the viral sialidase revealed that in the presence of calcium ions, influenza sialidase was greatly activated at a high temperature of 56°C (Fig 2). Sialidase activities of MuV and *S*. *pneumoniae* were also detected at 56°C (Fig 5). This is the first report showing that native sialidase on the viral surface is thermostable at a temperature of 56°C and that the temperature invoking its maximum enzymatic activity is much higher than 37°C, which is generally thought to be the optimal temperature for mammalian enzymes.

Sialidase activities of influenza virus strains are also known to have differences in low-pH stability in an acidic environment (pH 4.0–5.0) [51]. A/Puerto Rico/8/1934 and A/Memphis/1/1971 lost sialidase activity in an acidic condition of pH 4.0, while the activity of these two strains was stable even at 56°C (Fig 2). It is speculated that the steric structure of the influenza neuraminidase protein is affected by different mechanisms in low-pH and high-temperature conditions.

Results obtained from the reaction at pH 4.0 that are shown in Table 1 seem to be useful as a negative control for influenza detection and a proof for the presence of false-positives in a sample. However, it is considered that pH 4.0 is not suitable for a negative control in detecting pandemic IAV or avian IAV because all the pandemic IAVs and almost all the avian IAVs maintained their sialidase activity at pH 4.0, while almost all of the seasonal IAVs and IBVs lost their sialidase activity irreversibly at pH 4.0 [51]. Substrate stability in an acidic environment can be another problem. Under a pH 4.0 condition, an increase in the blank value occurs since the chemical binding between BTP3 and Neu5Ac is gradually cleaved and then fluorescent BTP3 is produced. If pH 4.0 is applied to the BTP3-based filter method, cleaved BTP3 would be deposited on a filter membrane and fluorescence could be observed even in the negative controls where viruses or *S*. *pneumoniae* are not present. For the above reasons, we considered that pH 4.0 is not suitable as a negative control for the detection of influenza viruses and as a confirmation of all of the false-positives in this method.

In the BTP3-based filter method, OV-resistant strains, A/Shizuoka/30/2014 and A/Shizuoka/1573/2009, showed highly fluorescent images in the presence of both OV and PV (Fig 5A). As expected, we were able to determine PV resistance in addition to OV resistance in these strains. Cross resistance against OV and PV was in good agreement with the results of a previous study showing that H275Y substitution neighboring the active site of IAV neuraminidase affects the interaction between the active site and a hydrophobic pentyl group commonly shared in OV and PV chemical structures [52]. PV-resistant viruses can also show resistance against ZV and LV because these NAIs have a guanidinium group that forms a strong salt bridge with glutamate residues at the 119 and 227 positions (based on amino acid numbering of human IAV N1 neuraminidase protein) [53,54]. Amino acid substitutions of these glutamates in combination with H275Y can confer a multi-drug resistance phenotype in IAV, but such cases are considered to be uncommon [28]. The IC_50_ values of OV and PV for A/Shizuoka/30/2014 were 1.7- and 1.9-fold higher than those for A/Shizuoka/1573/2009, respectively (S2 Table). Slightly stronger fluorescence of A/Shizuoka/30/2014 than that of A/Shizuoka/1573/2009 in the BTP3-based filter method with OV and PV seemed to be in agreement with the IC_50_ values of OV and PV (Fig 5A), indicating that the florescence image directly reflected the extent of drug resistance. Thus, the BTP3-based filter method can be used not only for screening of drug-resistant viruses but also for relative evaluation of IC_50_ values among drug-resistant viruses.

When human specimens are tested in the BTP3-based filter method, sialidase activities of MuV, *S*. *pneumoniae*, or HPIV-1 are predicted to be the main causes of false-positives for influenza virus detection. HPIV-1 is not a problem because of loss of its sialidase activity in the condition of pH 6.5 (Table 1). However, MuV and *S*. *pneumoniae* actually can become false-positive causes in the method. To discriminate influenza viruses from MuV and *S*. *pneumoniae*, we compared NAI inhibition patterns in the method among influenza virus, MuV, and *S*. *pneumoniae* (Fig 5B). MuV exhibited fluorescence for all of the NAIs, which showed no inhibitory effect on MuV. This might be due to the structural differences between influenza neuraminidase protein and MuV hemagglutinin-neuraminidase protein in accommodation of NAIs into the active sites. Together with such an MuV pattern in the BTP3-based filter method, the characteristic symptom of parotid swelling in MuV patients and epidemic information on MuV can also contribute to easy clinical diagnosis on MuV infection. *S*. *pneumoniae* was susceptible only to OV and fluorescence was observed in the presence of the other three NAIs. This could be explained by the fact that glutamate residues that interact with a guanidinium group in PV, ZV, and LV structures are not conserved in NanA, the prime neuraminidase of *S*. *pneumoniae*. In addition, the positively charged arginine residue at the 351 position of NanA extends further into the active site, thereby reducing the space available for the bulky guanidinium group [55]. These are the reasons why only OV inhibited *S*. *pneumoniae* sialidase activity. Since epidemics of a multi-drug-resistant influenza virus against all four NAIs or an influenza virus sensitive only to OV have not been reported in nature, for detection of currently prevalent influenza viruses, false-positives of MuV and *S*. *pneumoniae* can be ruled out by NAI inhibition patterns in the BTP3-based filter method. Abstracts of the interpretation of test results from the BTP3-based filter method are summarized in Table 3.

**Table 3:** Abstract of the interpretation of the test results in the BTP3-based filter method.

BTP3-based filter method	Positive control	OV	PV	ZV	LV	Negative control
Sample	(+)	(+)	(+)	(+)	(+)	(-)
NAI	(-)	(+)	(+)	(+)	(+)	(-)
<Results>						
NAIs-sensitive influenza virus	Positive	-	-	-	-	-
OV-resistant influenza virus	Positive	Positive	-	-	-	-
PV-resistant influenza virus	Positive	-	Positive	-	-	-
ZV-resistant influenza virus	Positive	-	-	Positive	-	-
LV-resistant influenza virus	Positive	-	-	-	Positive	-
OV- and PV-resistant influenza virus	Positive	Positive	Positive	-	-	-
ZV- and LV-resistant influenza virus	Positive	-	-	Positive	Positive	-
All four NAIs-resistant influenza virus	Positive	Positive	Positive	Positive	Positive	-
MuV	Positive	Positive	Positive	Positive	Positive	-
*S*. *pneumoniae*	Positive	-	Positive	Positive	Positive	-
HPIV-1	-	-	-	-	-	-
Absence of sialidase-containing viruses or bacteria	-	-	-	-	-	-

(+), presence of samples or NAIs; (-), absence of samples or NAIs; -, not detected.

In the absence of NAIs, the BTP3 fluorescence of *S*. *pneumoniae* was much weaker than that of MuV (first column in Fig 5B) even though the sialidase activity of *S*. *pneumoniae* showed 333% activation and that of MuV was decreased to 7.6% at pH 6.5 when compared with those in the pH 4.0 condition (Table 1). *S*. *pneumoniae* is known to possess cell wall-associated and secreted forms of NanA proteins [56]. In the pH optimization experiment (Table 1), both the activities of cell wall-associated and secreted forms of NanA proteins could be quantified by mixing an *S*. *pneumoniae* suspension with a BTP3-Neu5Ac solution in a microtube. On the other hand, in the BTP3-based filter method, only cell wall-associated NanA activity could be detected on the filter membrane because secreted NanA, the molecular size of which is about 100 kDa, can easily permeate 300-kDa molecular weight cut-off filters. In our preliminary experiments, almost all of the sialidase activity (98.7%) was detected in a flow-through sample of *S*.*pneumoniae* after 300-kDa molecular weight cut-off filtration. This means that only a small amount of cell wall-associated NanA was trapped on the filter membrane, which caused less efficient concentration of *S*. *pneumoniae* sialidase and resulted in a significant loss of fluorescence on the surface of the filter membrane.

ZV- and LV-resistant influenza viruses rarely emerge in nature. MuV and *S*. *pneumoniae*, which have no susceptibility to ZV and LV, can be discriminated from prevalent influenza viruses that are sensitive to ZN and LV. If a ZV- and LV-resistant influenza virus emerges in the future, such a mutant virus could also be differentiated from MuV and *S*. *pneumoniae* by both susceptibilities of OV and PV. In such a situation, the BTP3-based filter method can be a useful tool for detection of influenza viruses with OV and PV resistances or ZV and LV resistances. However, ZV- and LV-resistant mutation can be accompanied by PV resistance. In the case of an influenza virus resistant against three NAIs (PV, ZV, and LV) or all four NAIs, the BTP3-based filter method cannot differentiate these influenza viruses from *S*. *pneumoniae* or MuV.

To make sialidase detection with the BTP3-based filter method more specific to influenza virus, it is important to modify the Neu5Ac chemical structure to one that only an influenza sialidase can recognize. Introduction of a 4-guanidino group into Neu5Ac provides substrates with a highly specific property against influenza virus, and such a modification should also contribute to enhancement of the influenza specificity of the BTP3-based filter method. This has been confirmed by zanamivir, which is an Neu5Ac analogue with a 4-guanidino group. Indeed, ZstatFlu®-II, an influenza diagnostic kit that detects influenza sialidase activity, contains a 4,7-dimethoxy group in Neu5Ac of the substrate structure to improve influenza specificity [57]. We considered that modification of Neu5Ac in the BTP3-Neu5Ac structure is one of the effective means to achieve high specificity for influenza in the BTP3-based filter method. From this point of view, we have been studying another BTP3-based sialidase substrate with chemically modified Neu5Ac that is suitable for specific detection of influenza virus.

Commercial influenza immunochromatographic kits have been widely used in clinical practice for early diagnosis of influenza virus infection as POCT, providing results in 1 to 15 min [9]. The BTP3-based filter method can also provide test results within 15 min. Differential typing of IAV and IBV is possible in immunochromatographic kits. However, the clinical importance of IAV and IBV typing is unclear since NAIs are effective for both IAV and IBV. Although immunochromatographic kits are ready-to-use and involve an easy assay procedure without dedicated apparatuses, they have no ability to show viruses with resistance against NAIs. Besides, immunochromatographic kits are not suitable for high-throughput screening assays because the test device cannot handle a number of samples at the same time. The BTP3-based filter method would enable multiple sample handling and simultaneous detection of IAV and IBV with a single resistance against each NAI and double resistances against OV and PV or ZV and LV (Table 4).

**Table 4:** Characteristics of the BTP3-based filter method and a commercial influenza immunochromatographic kit.

Parameter	BTP3-based filter method	Commercial influenza immunochromatographic kit
Detection target	Sialidase	Viral nucleoprotein
Reaction time	15 min	1–15 min
Number of samples that can be tested simultaneously	Multiple	Single
Detection of drug-resistant and drug-sensitive influenza viruses	Yes	No
Differential typing of influenza A and influenza B	No	Yes

BTP3 fluorescence on the filter membrane is stable for a long time, making it possible to confirm test results of virus detection even several hours or days after termination of the sialidase reaction (S1 Fig). By directly visualizing viral sialidase activity, the BTP3-based filter method is expected to make contributions to the detection of drug-resistant viruses with unknown mutations and to the decision of what types of NAI would best respond to such mutants without the need to optimize specific antibodies or primers required for immunochromatographic kits or RT-PCR. In a pandemic of an influenza virus with novel drug-resistant mutations, reactivity of antibodies in immunochromatographic kits may not be guaranteed and acquisition of drug resistance cannot be determined by RT-PCR due to the lack of information on drug-resistant viruses except for N1 and N2 influenza subtypes. In the future, even if drug-resistant viruses with unknown mutations become prevalent, their NAI susceptibility profiles can soon be analyzed by the BTP3-based filter method and then appropriate therapeutic intervention can be taken at the early stage of a pandemic or epidemic. Simultaneous diagnosis of drug resistance and determination of antiviral strategies should aid in the establishment of selection criteria for truly effective therapeutic drugs.

The BTP3-based filter method involves neither expensive equipment nor advanced technological skill and can contribute to low-cost detection of influenza viruses with different NAI susceptibilities in a short laboratory turnaround time. In addition, this method is suitable for efficient analysis of numerous samples and is expected to be utilized in clinical and hygiene laboratories to differentiate and screen drug-resistant influenza viruses.

## Supporting information

S1 Fig Evaluation of long storage stability of BTP3 fluorescence. A/Puerto Rico/8/1934 (H1N1) was diluted with 100 mM acetate buffer (pH 6.5) supplemented with 50 mM CaCl_2_ to prepare a suspension of 2^4^ HAU and the sialidase activity was visualized by the BTP3-based filter method. Membranes of virus-loaded or blank samples were kept in the dark for 28 days. BTP3 fluorescence just after (Day 0) and 28 days after the BTP3-Neu5Ac reaction was visually compared under UV irradiation with a handheld UV flashlight at 375 nm.
(TIF)

S1 Table Viral sialidase activity after ultrafiltration with molecular weight cut-off filters. A/Puerto Rico/8/1934 (H1N1) was diluted with 100 mM acetate buffer (pH 6.5) supplemented with 50 mM CaCl_2_ to prepare a suspension of 2^3^ HAU. BTP3-Neu5Ac was diluted in 100 mM acetate buffer (pH 6.5) supplemented with 50 mM CaCl_2_ to give a concentration of 200 μM. One hundred microliters of the virus suspension was loaded onto centrifugal filter units with different pore sizes (Nanosep® 30K Centrifugal Filter Device, Nanosep® 100K Centrifugal Filter Device, and Nanosep® 300K Centrifugal Filter Device, PALL Co., NY, USA), followed by centrifugation at 2,000 *g* for 1 min with the benchtop centrifuge. After centrifugation, an equal volume of flow-through and 200 μM BTP3-Neu5Ac in 100 mM acetate buffer (pH 6.5) supplemented with 50 mM CaCl_2_ were mixed in the microtube and incubated at 56°C for 15 min with the dry heat block. One hundred microliters of the reaction mixture was transferred to a 96-well microtitre plate, and fluorescence from sialidase reaction was quantified by the microplate reader. Sialidase activity (%) in the flow-through samples was expressed as a percentage of the enzymatic activity of 2^3^ HAU A/Puerto Rico/8/1934 (H1N1).
(XLS)

S2 Table Sialidase inhibition assay of NAIs. Four clinical isolates, A/Shizuoka/17/2016 (H1N1pdm), A/Shizuoka/19/2016 (H1N1pdm), A/Shizuoka/30/2014 (H1N1pdm) and A/Shizuoka/1573/2009 (H1N1pdm), were standardized to give equivalent sialidase activities. Forty microliters of the virus suspension in PBS was mixed with 5 μL of ten-fold dilutions of NAIs or distilled water alone on a 96-well microtitre plate. The mixture was incubated at 37°C for 20 min. Five microliters of 1 mM 4MU-Neu5Ac was added on ice and then incubated at 37°C for 60 min. Enzymatic reaction for 4MU-Neu5Ac was stopped by 50 μL of 100 mM sodium carbonate buffer (pH 10.7). Fluorescence from sialidase reaction was quantified by the microplate reader with wavelengths of excitation and emission at 355 nm/460 nm for 4-metylumbelliferone. The concentration of NAI that reduced viral sialidase activity by 50% relative to a control mixture without NAI was determined as IC_50_. Percent inhibition of the sialidase activity at respective NAI was plotted against NAI concentration, and IC_50_ values of NAIs were calculated using GraphPad Prism 5 software (GraphPad Software, CA, USA). OV, PV, ZV, and LV represent oseltamivir, peramivir, zanamivir, and laninamivir, respectively.
(XLS)
