# Supplementary figures and images for: An easy, rapid, and sensitive method for detection of drug-resistant influenza virus by using a sialidase fluorescent imaging probe, BTP3-Neu5Ac
Source: PLoS One. 2018 Jul 12;13(7):e0200761. doi: 10.1371/journal.pone.0200761 (PMC6042793; doi:10.1371/journal.pone.0200761)

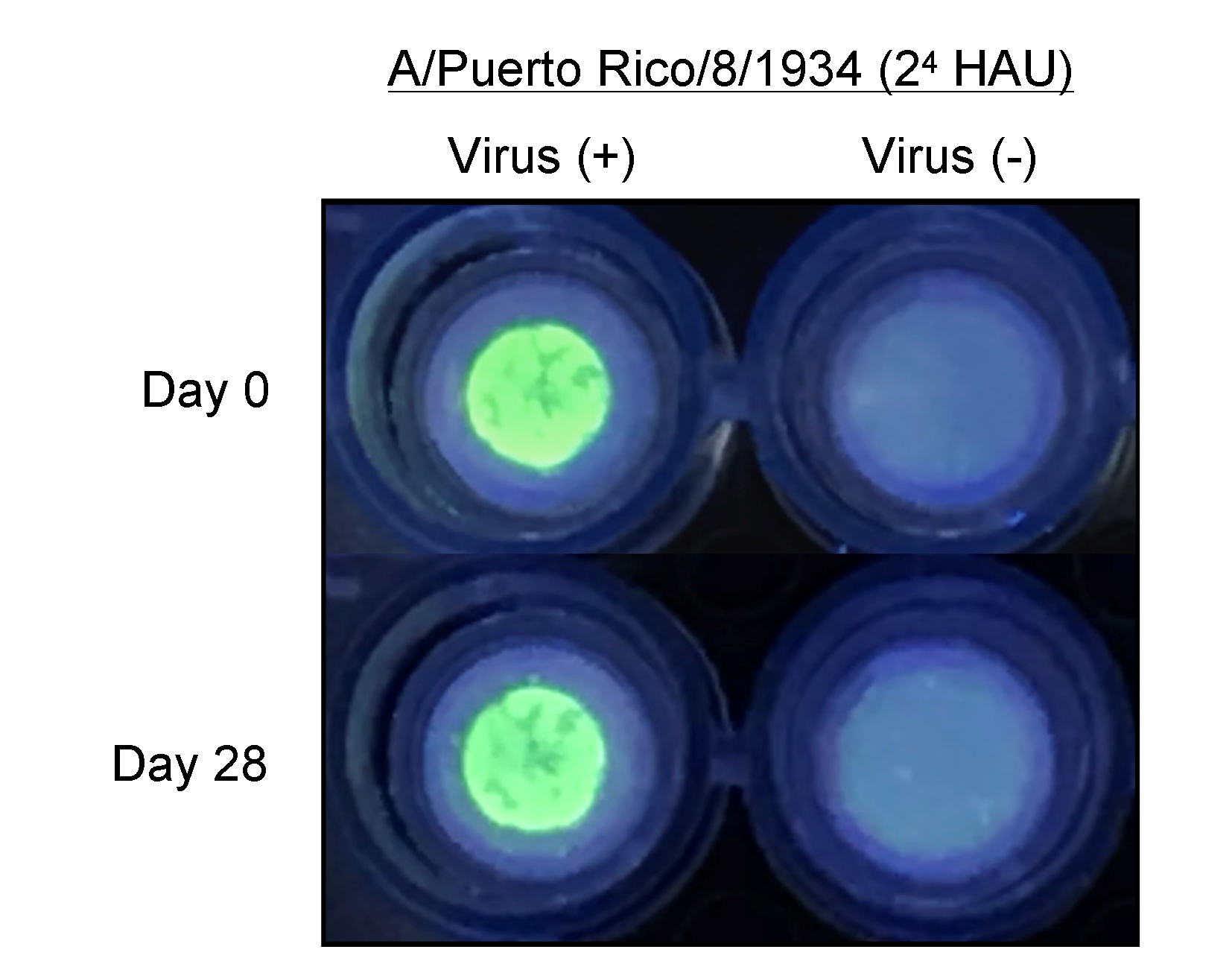

Supplement: S1 Fig — A/Puerto Rico/8/1934 (H1N1) was diluted with 100 mM acetate buffer (pH 6.5) supplemented with 50 mM CaCl2 to prepare a suspension of 24 HAU and the sialidase activity was visualized by the BTP3-based filter method. Membranes of virus-loaded or blank samples were kept in the dark for 28 days. BTP3 fluorescence just after (Day 0) and 28 days after the BTP3-Neu5Ac reaction was visually compared under UV irradiation with a handheld UV flashlight at 375 nm. (TIF) [file pone.0200761.s001.tif]
